# Genes and genomes and unnecessary complexity in precision medicine

**DOI:** 10.1038/s41525-020-0128-1

**Published:** 2020-05-04

**Authors:** Rama S. Singh, Bhagwati P. Gupta

**Affiliations:** Department of Biology, Origins Institute, McMaster University, 1280 Main Street West, Hamilton, ON Canada

**Keywords:** Molecular medicine, Genome-wide association studies, Risk factors

## Abstract

The sequencing of the human genome heralded the new age of ‘genetic medicine’ and raised the hope of precision medicine facilitating prolonged and healthy lives. Recent studies have dampened this expectation, as the relationships among mutations (termed ‘risk factors’), biological processes, and diseases have emerged to be more complex than initially anticipated. In this review, we elaborate upon the nature of the relationship between genotype and phenotype, between chance-laden molecular complexity and the evolution of complex traits, and the relevance of this relationship to precision medicine. Molecular contingency, i.e., chance-driven molecular changes, in conjunction with the blind nature of evolutionary processes, creates genetic redundancy or multiple molecular pathways to the same phenotype; as time goes on, these pathways become more complex, interconnected, and hierarchically integrated. Based on the proposition that gene-gene interactions provide the major source of variation for evolutionary change, we present a theory of molecular complexity and posit that it consists of two parts, necessary and unnecessary complexity, both of which are inseparable and increase over time. We argue that, unlike necessary complexity, comprising all aspects of the organism’s genetic program, unnecessary complexity is evolutionary baggage: the result of molecular constraints, historical circumstances, and the blind nature of evolutionary forces. In the short term, unnecessary complexity can give rise to similar risk factors with different genetic backgrounds; in the long term, genes become functionally interconnected and integrated, directly or indirectly, affecting multiple traits simultaneously. We reason that in addition to personal genomics and precision medicine, unnecessary complexity has consequences in evolutionary biology.

## Introduction

Throughout medical history, the disciplines of embryology, anatomy, and physiology have provided the biological picture of the human body and the basis for detecting diseases and devising treatments. Despite heightened interest in blood chemistry at the turn of the nineteenth century, as late as the 1980s, genetics was not a required subject for students preparing to enter the medical profession. Indeed, genetics had a limited role in the human health professions even long after the discovery of the structure of DNA. The sequencing of the human genome in the 1990s heralded the new era of ‘molecular medicine’ and raised expectations that personalized/precision medicine would promote long and healthy lives. Recent population genomics studies have dampened this expectation; the relationships among mutation/risk factors, molecular complexity, and disease are apparently far more complex than originally presumed. Hence, two individuals bearing the same set of risk factors may not present (or exhibit the symptoms of) the same disease. This is the kind of problem that precision medicine is expected to overcome.

The lack of genetic determinism in disease is the result of molecular contingency, i.e., molecular constraints and historical contingency. This very much resembles the problem of chance and necessity or contingency and fate in evolutionary biology. Whether evolution is deterministic, i.e., predictable and repeatable, or contingent and unpredictable is an interesting topic in evolutionary biology^1^ (for a recent review see ref. ^2^). Evolutionary contingency can result from differences in initial conditions or from historical and environmental events. It can also occur due to genetic contingency; for example, sequential mutations that are unavailable in requisite order^2^. Comparative experimental studies show that phenotypic evolutionary ‘repeatability’ is common when the founding populations are closely related, perhaps resulting from shared genes and developmental pathways, whereas different outcomes become more likely as historical divergences increase^2^. Applying this finding at the molecular level, we predict that two individuals sharing the same risk factors should share a disease; however, in reality, this does not occur.

A Personal Genome Project Canada study^3^ using a sample of 56 whole genome sequences reported that 94% (53/56) of individuals carried at least one disease-associated allele (mean 3.3/individual); 25% (14/56) had a total of 19 alleles with other health implications and an average of 3.9 diplotype associated with risk for altered drug efficacy or reactions. Of the 19 health-implicated variants, 6 were ‘pathogenic or likely pathogenic,’ some of which caused no adverse symptoms in the carriers.

While there might be an apparent contradiction between the micro and macro levels of biological organization, none exists. Just as, at the macro level, diverging developmental pathways lead to different outcomes between species, so, at the micro level, alterations in developmental pathways can lead to non-concordance between individuals sharing the same risk factors. In other words, the discordance between the molecular and morphological levels is the result of differences in hierarchical complexity and evolutionary integration. In an insightful paper, ‘Evolution and Tinkering,’ Jacob^4^ pointed out the contingent nature of evolution, permeated by constraint, historical circumstances, and the blind nature of evolutionary processes, and likened natural selection to a tinkerer as opposed to an engineer. Unlike the engineer, evolution makes use of whatever molecular material exists at the time. It does not lead to perfection and it has no plan; in other words, it is blind. Evolutionary blindness amplifies evolutionary contingency.

In this article, we focus on the blind nature of evolution and its effects on molecular complexity. We propose a theory of molecular complexity, consisting of necessary and unnecessary complexity in living systems. We first argue that the exceedingly high level of unnecessary complexity in the biochemical, molecular, and developmental pathways of organisms has more to do with the blind process of evolution than with the randomness of mutations. In other words, the fundamental limitation of the evolutionary process is that it does not pick and choose genes during generational transition; rather, it functions by integration and builds on the previous step in the selection process in each subsequent generation. Thus, the starting condition constrains what may be possible in the future and, therefore, is the basis of the mind-boggling, incredible, and even excessive and unnecessary complexity of underlying biological processes in living systems. We argue that the concept of necessary and unnecessary complexity is relevant to current discussions on genomics and precision medicine. Unnecessary complexity implies genetic redundancy and can explain why two individuals with the same risk factor may not always show symptoms of the same disease, possibly due to underlying differences in their genetic backgrounds and gene interaction networks. The molecular network around each risk factor is subject to evolutionary contingency, making precision medicine uncertain, chance-ridden, and probabilistic.

## Complexity in living systems

Complexity can be defined as the degree of interactions, ordered or disordered, between the components of a system. In the physical world complexity peaked at the end of the formation of physical bodies—planets, stars, and galaxies. In contrast, in the living world, molecular complexity, for simplicity defined as the total number of gene-gene/molecular interactions summed over the life of the organism in its natural environment, has been increasing over time. Evolutionary theory posits that organismic complexity and diversity is the result of the transformation of random mutations into non-random functional complexity of the organisms by natural selection on the basis of said mutations’ effect on survival and reproduction^4–6^. An outstanding feature of all organisms is their complexity and as Mayr^7^ puts it, ‘every organic system is so rich in feedbacks, homeostatic devices, and potential multiple pathways that a complete description is quite impossible.’

Complexity in living systems cannot be fully understood unless we understand the intricate nature of how selection acts at the level of genes. Biological complexity is the result of gene interactions and context, combined with blind ‘evolutionary walk.’ The longer the walk, the greater the complexity, as evidenced by the incredibly diverse forms seen in multicellular organisms. The role of chance is still misunderstood in biology, as there is little appreciation of how the exceedingly slow processes of new mutations and incremental natural selection combine to operate in the blind evolutionary process of population turnover from generation to generation. It is more than the chance fixation of an allele; chance is part and parcel of gene interactions.

The task of natural selection can be understood at three levels: (1) in population genetics, natural selection operates on the basis of average fitness of a gene over all genotypes (haploid gametes, diploid genotype, and mating pairs) and all environments in which it is found, acting on what Fisher called the net ‘reproductive value’ of a gene^8^; (2) in ecology, one way of measuring selection is in terms of the intrinsic rate of increase in the number of individuals in a population^8^; and (3) finally, in molecular biology, natural selection is strictly associated with the gene and the rate of evolution is ‘projected’ through the balance of dichotomized ‘good genes’ and ‘bad genes’ or advantageous, deleterious, and neutral^9^. The language of molecular biology is deterministic (e.g., wild type, mutant type, deleterious mutation, promoter, enhancer, activator, repressor, etc.) and gives the impression that these phenomena are inherent to the gene and free of external forces or environmental perturbations; but this is not entirely true. For example, take the case of transcription factors that bind to specific enhancer DNA sequences of target genes. The combination of transcription factor recognition sequences, their locations within the enhancer regions of genes, and the numbers of binding sites are all dictated by the sequence of the gene. However, interaction between a transcription factor and a given binding site is variable, due to changes in the nuclear microenvironment. The overall transcriptional response can be consistent and robust due to a higher level of regulation^10^.

Another way of understanding the complexity of natural selection is to consider the nature of the interaction between genes and environment in the organism. As demonstrated by Clausen, Keck, and Hiesey^11^ using transplantation experiments with climatic types from the plant genus *Achillea* (yarrow), each organism is a ‘genotype’. While average fitness may capture the performance of a gene, it does not capture the idea that gene–gene interactions themselves are subject to change in a variable environment. As we reported previously^12^, ‘Waddington’s genetic assimilation^13^, Lerner’s genetic homeostasis^14^, and Schmalhausen^15^, Dobzhansky^16^ and Lewontin et al.’s^17^ norm of reaction, in different ways, all point to the same facts: (i) that organisms are internally heterogeneous and open systems sustained by influx of energy, (ii) that biological information necessary for the organism’s development and function is not entirely contained in the DNA sequences but is also distributed across the cellular matrix of the egg and the tissues, (iii) that developmental information is built into hierarchical cellular subsystems, and (iv) that evolutionary information is distributed in the patterns of organism–organism and organism–environment interactions.’ The reaction norm of a genotype, i.e., the phenotypic expression of the trait over a range of environments, is not fixed, but instead is dynamic and subject to change based on the interaction between genes and the environment.

## Is complexity necessary?

It may seem simplistic, but it is important to point out that, contrary to statements by the creationists, evolution is not a random process and that complexity is the outcome of evolution. Mutations occur randomly; evolution does not. Evolution, driven by natural selection, is a process that favors genotypes or organisms which show higher potential to compete, survive, and reproduce. Organisms do not wait for new mutations to adapt to the changing conditions of life. In the long term, new mutations arise and provide organisms with new genetic potential and opportunities to make use of the environment in new ways. In the short term, however, generation by generation, organisms make use of the genetic variation that is already present in their genomes. Moreover, there is a lot of genetic variation in populations^18^.

Furthermore, contrary to common misperception, mutations are not segregated into two categories—good and bad. While most new mutations that arise at every generation are expected to be deleterious and therefore eliminated, a few rare beneficial ones that have managed to survive random elimination may persist and spread throughout the population. The rest, which represent the majority and grade from mildly deleterious to neutral to slightly beneficial, persist in populations. These are the mutations that provide the basis for evolutionary change^9,19^.

It may not sound assuring that mildly deleterious, slightly beneficial, or even neutral mutations make up the bulk of the variation that is the basis of adaptive change, but it works because natural selection has some unique features that are generally underappreciated. First, barring lethal mutations, selection does not operate on one mutation at a time. Instead, it acts on all of them together, i.e., at the level of an organism’s genome or its genotype. Second, in the middle range of the fitness spectrum, effects are not constant, but instead vary based on cells/genomes. Gene–gene interaction and context (i.e., cell, tissue, developmental stage) are of prime importance (for examples, see refs. ^18,20–22^). Third, genes are not a bag of free marbles; linkage plays a major role in determining the fate of mutations that are beneficial, deleterious, or neutral. Beneficial mutations drag their neighbors (hitchhikers) with them, as do deleterious mutations. The same is true with highly beneficial mutations: when they sweep through the population, they drag their neighbors with them^23^. Fourth, barring the effects of linkage, even the best interacting gene combinations change from generation to generation due to sexual mixing. Mutations that are good interactors—what evolutionary biologist Ernst Mayr termed ‘good mixers’—remain, while the rest move on^24^. Finally, because natural selection is blind, i.e., it only works on the fitness of the existing genotypes in the present environment, with no consideration for the future, it results into two kinds of gene-gene interactions or complexity: necessary and unnecessary.

By necessary complexity at the molecular level, we mean the minimum number of gene-gene interactions and minimum biochemical path lengths necessary for a given molecular function, trait or an organism. However, because of linkage, new mutations, new interactions, and, most importantly, the blind nature of the selection process, evolution necessarily leads to the creation of extra, repetitious interactions of a roundabout nature, crisscrossing existing biochemical pathways and increasing the length of the molecular pathways. This is unnecessary complexity. Here, the term ‘unnecessary’ refers to a particular biochemical pathway and does not apply to any future usefulness of the complex pathway in a changing environment. It is important to point out that unnecessary complexity should not be considered superfluous ‘noise,’ but is rather an integral part of the evolutionary process and of the genome itself.

It is worth mentioning that several of our colleagues have suggested alternative terms for unnecessary complexity, for example, redundancy, excess complexity, chance complexity or unpredictable complexity. Our choice, i.e., unnecessary complexity, is not perfect but it implies what we want to convey, that it is an unwanted evolutionary baggage due to ever increasing and accumulating molecular interactions and long molecular pathways. It does not mean that unnecessary complexity has no effect on the operation or the outcome of natural selection now or in the future.

## Evolution of complexity: genic, genomic, and developmental

Studies in arthropods have led to major insights into the complexity of developmental mechanisms and evolutionary changes. Experiments on the fruit fly *Drosophila melanogaster* have uncovered the complexity of gene interaction networks during early development^25,26^. For example, the earliest set of genes that are activated in the embryo, termed the ‘maternal’ class of genes, help establish body axes. Subsequent to the formation of body plan, segments and polarities of segments require the function of genes belonging to zygotic, gap, pair-rule, and segment polarity classes^27^. The cooperative and antagonistic actions between these genes ensure a precise and robust sequence of developmental events in the embryo, leading to the formation of tissues and organs at later stages. While the entire developmental process encoded in the DNA sequence is a necessary component of evolution, the individual mutations involved are not uniquely necessary; they can be replaced with others. Genomic and proteomic studies are providing insight into the old question of developmental constraints in evolution. Recent studies have shown that developmental constraint and selection work together: development can constrain evolution in the short term, but selection can alter and reshape those constraints in the long term^28,29^. While developmental constraint on genes affecting embryology is not unexpected, as shown by Artieri and Singh^30^ using patterns of gene expression during *Drosophila* ontogeny, it is not development but Darwinian ‘selection opportunity’ that dictates post-embryological diversification^4,30,31^.

Technological advancements over the last decade have made efficient large-scale genome sequencing of organisms easily available. The analysis of sequence data has revealed the structure of genes, gene families, and their chromosomal organizations (e.g., see www.genecards.org, www.informatics.jax.org, www.flybase.org, and www.wormbase.org). Genomic data together with gene expression studies are providing insight not only into the history of evolution but also on the type and extent of standing variation in populations. Some of the highlights reported by these studies are summarized below.

### Number of genes do not correlate with complexity

While higher organisms have more protein coding genes, variation in gene number does not strongly correlate with morphological complexity. For example, the nematode *C. elegans* has more genes than the fruit fly *D. melanogaster*, but the latter has appendages and is morphologically more complex. Protein-coding genes in humans, excluding splicing variants, are converging toward 20,000, even though the entire genome is predicted to code over 200,000 transcripts^32^. In addition to mRNA and proteins, there are increasing numbers of non-coding RNA transcripts in metazoan genomes such as micro RNA (miRNA) and long non-coding RNA (lncRNA)^33^. In humans, there are more non-coding RNA genes than protein-coding genes^32^.

### Evolution occurs by making alternate use of genes

Evolution occurs by making alternate uses of existing genes through structural^34–36^ and regulatory changes^37,38^. This is reflected in the 99% sequence similarity shared by humans and chimpanzees, with only 6% of the genes in one species lacking a known homolog in the other^39^. Despite such a high level of sequence conservation, about 80% of proteins in humans and chimps differ in at least one amino acid^35^ and 10% of genes between humans and chimpanzees differ in their expression in the brains of the two species^40,41^.

### Number of genes affecting a trait appears large

The notion of candidate genes/loci persists and guides much of health genomics for practical reasons. Studies involving the mapping of quantitative trait loci (QTL) have shown that, directly and indirectly, traits are affected by a large number of genes^42,43^. As an example, early studies of variation in human height initially implicated half a dozen to a dozen genes. A recent genomic meta-analysis of human height variation involving over 700,000 individuals has detected over 3290 significant SNPs^44^. Yet together, these SNPs may account for only 24% of the variance in height. The same is largely true for all complex diseases. Genomics is driving home the lesson that there are protein-coding and non-coding genes that perform a variety of functions, but there are no genes specific for a trait. Genome-wide association studies (GWAS) have led to the identification of genes linked to specific traits and diseases^45^ (https://www.genome.gov/about-genomics/fact-sheets/Genome-Wide-Association-Studies-Fact-Sheet). The data reveal that genes are shared between traits. A recent paper by Boyle et al. presents an ‘omnigenic’ model of complex traits^46^, proposing that all genes expressed in disease-relevant cells are involved in a functional network and hence contribute to the condition.

### A significant part of non-coding DNA may be involved in regulation

The ENCODE project (Encyclopedia of DNA Elements; the ENCODE Project Consortium 2012) initially reported a large proportion of the genome to be functional, but ultimately scaled it down to approximately10%. This added to the ‘junk DNA’ debate and questions regarding the proper biological function of a gene^47,48^. Although a large proportion of mammalian DNA may have no necessary or essential function, this should not be interpreted as lacking in function or being inert. Such apparently ‘non-functional’ DNA may be part of the unnecessary complexity of the uncommitted ‘gene pool’—part of current phenotypic plasticity devoid of teleological explanation for future use.

### Phylogenetic gene complexity shows the same function can be shaped by different genes

Recent genomic studies of protein evolution in anatomical traits of *D. melanogaster* embryos showed that younger genes, i.e., genes that are comparatively newer based on phylogenetic analysis, had lesser tissue distribution, fewer interactions, high expression levels, and less evolutionary constraint^49,50^. Given that the function of a gene is not fixed and functions evolve between genes as well as within genes over time, we can expect the complexity of interaction networks of newer genes to increase with time. In a study of adaptation in protein-coding gene trees in the primate clade, Daub et al.^51^ remarked: ‘several gene sets are found significant at multiple levels in the phylogeny, but different genes are responsible for the selection signal in the different branches. This suggests that the same function has been optimized in different ways at different times in primate evolution.’

### Evolution by gene regulation is not ‘break free’

In the post genomic world, the old ‘major vs. minor’ or ‘regulatory vs. structural’ mutation debate has been restructured and refined in terms of the role of *cis*-regulation vs. structural mutation in evolution^52–54^. Mutations in ‘*cis*’ elements generally affect the expression of individual genes, possibly contributing to regulatory evolution^54^. However, there are also examples of stabilizing selection operating on gene expression that tends to compensate for ‘*cis*’ changes (e.g., see ref. ^55^), leading to the evolution of biological complexity. While new cases of evolution by *cis*-regulatory mutations are being discovered, they are still far fewer than those by coding mutations^52,53^. Although the importance of *cis*-regulatory mutations in evolution is well documented, the real question involves neither their crucial role nor their unique contribution to the evolution of morphology. Instead, it is whether *cis*-regulatory mutations provide a source of variation that, unlike protein-coding mutations, is potentially large and pleiotropy-free, i.e., have no deleterious side effects and provide possibilities for ‘break-free’ evolutionary change. It is erroneous to argue that, unlike protein-coding variation, *cis*-regulation variation is free of pleiotropic effects or free of constraints^56,57^. Molecular population genetic studies inform us that genetic variation is not the limiting factor in evolution; the limiting factor is ‘selection opportunity’^58^. Evolution does not work toward producing perfect proteins. The protein-protein interactions and any negative effects arising therefrom are part of the genetic machinery involved in evolutionary change. Negative pleiotropy in structural mutations may not be any worse than the negative effect of gene expression in an unwanted place and time^53^. Negative pleiotropic effects of structural mutations are factored into the rate of evolution through compensatory mutations and gene-gene interactions. Similarly, *cis*-acting regulations are obviously important in controlling gene expression and may appear to provide a limitless rate of evolutionary change; however, we do not need to argue that evolution in nature is slow and incremental.

### Molecular redundancy is a universal feature of organisms

**Fig. 1:**
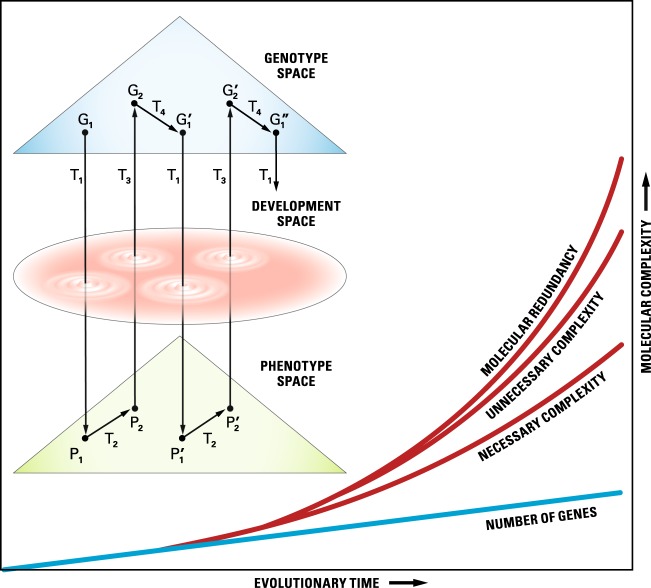
Schematic representation of genotype-phenotype transformation from one generation to the next. G and P are the spaces of the genotypic and phenotypic description. G_1_, G′_1_, G_2_, and G′_2_ are genotypic descriptions at various points in time within successive generations. P_1_, P′_1_, P_2_, and P′_2_ are phenotypic descriptions. T_1_, and T_3_ are laws of transformation from genotype to phenotype and back, respectively, during development. T_2_ are laws of population biology, and T_4_ are laws of Mendel and Morgan about gamete formation. Necessary and unnecessary complexities and molecular redundancy are defined in the text. (After Lewontin^19^). The graph lines are not intended to mean monotonic increase.

Organisms are both the subject and the object of evolutionary change. Since the organisms’ environment is not constant, we can expect some degree of molecular flexibility in the ability of the organisms to adapt to environmental fluctuations experienced over their lifetime. Such a flexibility could come from at least three distinct but interrelated sources. One of these is what we have termed as unnecessary complexity, i.e., multiple redundant gene interactions and pathways. The second source of flexibility is over-expression of genes or up-regulation of pathways. It is expected that the functional integrity of any pathway/network would be limited by the least-expressed genes and such genes may be under pressure to be upregulated. Any increase in gene expression will contribute to higher probability of random molecular interactions thereby forming the basis of new functions and, therefore, new evolutionary adaptations. The third source is gene-environment interactions, termed ‘norm of reaction’^17^. The unnecessary complexity together with molecular flexibility is what we have termed as molecular redundancy (Fig. 1).

## Unnecessary complexity in precision medicine

The implications of personal genomics to precision medicine are based on information obtained from individual genomes regarding genes/alleles and the associated risks, if known, of developing a disease. The genomic approach will work for variants that cause discrete and large phenotypic effects but not for variants of small effects associated with varied phenotypic spectrum or influenced by the environment^3^. This raises the question: where do our ideas of genes with large vs. small effects, major vs. minor genes, and Mendelian vs. quantitative traits come from?

The reigning paradigm of genetics has been the dichotomous division of phenotypic traits into discrete/monogenic/Mendelian and continuous/polygenic, i.e., traits governed by a few major, large effect, genes and traits governed by many, small effect, genes, respectively. Mendel was the originator of large effect genes as he deliberately chose traits with discrete and large effects^12^. Karl Pearson is known for continuously distributed traits^59^. As evidence accumulated, some room was made for Mendelian traits (associated with genes having large effects) to be affected by polygenes and polygenic traits. These two classes of genes and traits have been taught for over a century and research programs in genetics are based on this dual categorization. Recent genomic studies including GWAS have relied on this mono-poly-paradigm and are meant for finding disease-causing genes with large effects. For example, a study by Reuter et al.^3^ uncovered a total of 172 deleterious mutations with small effects and 19 mutations with health implications using a sample of 56 genomes,. The analysis was done one gene at a time and did not consider gene-interaction networks. The authors pointed out that, ‘this approach will continue to be appropriate for genetic variants with substantial discrete impact on phenotypes. It should be emphasized, however, that the full impact of personal genomics in precision medicine will emerge as we recognize that the variants with incremental effects and complex interactions are all influenced by recent human adaptations.’

The investigation of variants with small effects are more problematic. They could be studied, in principle, by increasing the sample size. The polygenic risk score (PRS), based on large-scale genomic studies, offers effective control of the phenotypic spectrum and holds the potential to revolutionize genetic studies of quantitative traits controlled by polygenes^60,61^. Large-scale genomic studies promise to unravel the ultimate nature of genetic variation including additive, gene–gene, and gene–environment interaction effects^60^.

The paper by Boyle et al.^46^ about the omnigenic model of complex traits further adds to the complex relationship between personal genomics and precision medicine that challenges the polygenic model of quantitative traits. Using human height as an example, the authors show that contrary to the traditional explanation involving a dozen polygenes with small effect alleles, the trait is affected by many genes. They state that, ‘Putting the various lines of evidence together, we estimate that more than 100,000 SNPs (single nucleotide polymorphisms) exert independent causal effects on height, similar to an early estimate of 93,000 causal variants based on different approaches.’ In the end, this led the authors to propose that regulatory networks are interconnected in such a way that all genes expressed in disease-relevant cells are able to exert their effect on core disease-related genes.

The omnigenic model of complex traits amounts to a call for a paradigm shift. This model relates to the relative number of genes affecting a trait or disease and does not do away with the paradigm that traits/diseases may have core genes of large effects as well as many genes of small effects^62^. In the following discussion, we present various lines of argument in favor of the omnigenic model and then propose that unnecessary complexity is the reason why ‘core genes are highly outnumbered by peripheral genes and that a large fraction of the total genetic contribution to disease comes from peripheral genes that do not play direct roles in disease’^46^.

First, genomic studies have shown that there are simply not enough genes to provide for trait-specific core genes. Therefore, core genes must be shared between traits. Also, for rapidly evolving organs such as the human brain, core genes are more likely to be shared between different functions than in other organs. Second, developmental studies have shown that development takes place by sequential, time-dependent command sequences. This means that genes at the lower levels of development are expected to affect genes and traits that develop at higher levels/later stages. Third, population genetics models of quantitative traits emphasize evolution by shifting allele frequencies at large number of underlying genes. Fourth, in the network model of gene action, functional integrity of the hub^53^ will be governed by levels of gene expression in that hub. Genes shared between overlapping pathways may be required in varying amounts at different points and, therefore, as an evolutionary strategy, in the absence of gene-by-gene tuning, it may be better to make those genes expressed at the highest levels they are needed in that network of pathways. This may lead to over expression of genes at times and in places where they are not always needed. This would explain the presence of large amounts of phenotypic flexibility so commonly observed in nature^63^. Finally, the ever-increasing and permanent nature of unnecessary complexity assures that trait-specific networks in general cannot become isolated and specialized at any levels of cellular organization and will be shared between network hubs and traits. Unnecessary complexity may be a curse for precision medicine, but it is a boon for the evolutionary process as it provides infinite ways of traversing the evolutionary landscape. In a way it is also a boon for carriers of risk factors as carrying risk factors does not doom you to the disease.

## Unnecessary complexity and the etiology of cancer

Unlike hereditary diseases, cancers caused by somatic mutations form a special category. Cancer biology can make use of evolutionary principles of clonal reproduction and evolution. Both mutation-centric and context-based selection mechanisms, based on population divergence, have made home in the emerging field of cancer genomics^64^. Study of mutation or gene expression variation within and between tumors and their association with the disease phenotype is like the study of variation within and between clonal populations. The major difference is that unlike natural clones or clonal organisms, tumors represent transformed cells with DNA complement of a complex organism. In cancerous cells one can expect reductive evolution through genes loss^65^ and epigenetic variation in gene expression, and therefore differentiating between ‘driver’ and ‘passenger’ mutations may not always be easy. Cancer genomics provides a unique opportunity for exhaustive investigation of gene expression and gene interaction network in organ-specific tumors to be able to pinpoint genes or groups of genes involved in the disease^66^. Unnecessary complexity implies that barring cases of strongly inherited cancers we should expect multiple genes and multiple, overlapping or non-overlapping, gene interaction networks not only to be involved between tumors of different organs, but even between different tumors of the same organ^67,68^. Cancer genomics linked with integrative studies stand to provide basic knowledge about the ‘functional genome’ which not only will aid in finding a cure for the disease but also, as a side benefit, enrich our understanding of the genotype-phenotype space (Fig. 1).

## Unnecessary complexity beyond precision medicine

Unnecessary complexity has relevance beyond precision medicine. We provide two examples. One area is evo-devo. Ever since Goldschmidt^69^ argued for the role of major, regulatory mutations as the material basis of evolutionary change (above species level), evolutionary developmental biologists have inherited the regulatory framework and have presented alternate mutation-centric theories of evolution. They have done it by ignoring the role of standing population (genetic) variation which is predominantly fine grained, by assuming a hard fitness landscape, by ignoring the role of gene-environment interactions and norm of reaction in altering the fitness landscape, and by treating evolution as a transition between robust phenotypes, character states, through mutations with predetermined, fixed fitness function (e.g., see refs. ^70–72^). Whether fitness landscapes are ubiquitous and are smooth or rugged is an open question^73^. Unnecessary complexity, as we have described here, is precisely the result of the factors that evo-devo tends to ignore in its blueprint for evolutionary change. We propose that genetic variation generated by gene-gene interaction is a greater source of variation for evolutionary change than structural variation or regulatory gene mutations (Fig. 1).

A second area of application is the nature of the profound differences between the physical and the biological view of the world or, in other words, why biology is not physics^74^. Genotype-phenotype relationship can be visualized at two levels: at the level of single genes as in Mendelian genetics and precision medicine, and at the level of the organisms during ontogeny and development. In recognition of the conceptual contribution of Erwin Schrodinger to the foundation of molecular biology, and of Richard Lewontin through his life-long battle to show the importance of “interaction and context” in the development of phenotype, and in interest to start dialog on why there are no laws of Biology, we propose that genotype-phenotype space be named as ‘Schrodinger-Lewontin Space’. Evolution in Schrodinger -Lewontin Space over time gives rise to the ‘Darwinian Space’, i.e., the space occupied by living beings in space and time.

Unnecessary complexity dampens the hope that the laws of biology, if there are any, will be explained in a straightforward manner by applying the laws of physics and chemistry, however, evolution of molecular complexity provides a common ground for dialogs between physicists and biologists. Molecular complexity guarantees evolutionary directionality and adds to the profound wonder of life by reminding us, Max Delbruck pointed out, that ‘any living cell carries with it the experiences of a billion years of experimentation by its ancestors’^75^.

## Conclusions

Unnecessary molecular complexity is the combined result of constraints, historical circumstances, and blind evolution. Unnecessary complexity is not simply ‘noise’ but rather constitutes part of an organism’s hierarchically integrated genetic system. Natural selection acts on phenotypes that do not have a one-to-one relationship with genotypes. All genes, good or bad, major or minor, reside in the same cell and depending on the molecular contingency (i.e., the availability of particular sets of mutations, gene–gene interactions, and sampling effects), the same mutation will end up with different gene partners in different individuals or organisms. Given that organismal fitness depends on the function of all genes and on how they interact, mutations with significant deleterious effects would either get eliminated or persist in populations due to gene interactions and functional compensation by other genes. Gene interaction and compensatory evolution would lead to the persistence of many deleterious genes beyond their life expectancy. What this means is that depending on the context, the same mutation can be deleterious in one individual, have a mild effect in a second, and no effect whatsoever in a third. Interaction and context are of the essence^18^. Hence, two individuals may have the same set of deleterious mutations but may experience different health outcomes. Unnecessary complexity is going to make precision medicine more challenging and more important at the same time. An appreciation of evolutionary principles and mechanisms can only help (Table 1).

**Table 1:** A list of key terms and definitions (source cited).

Evolution a blind process: Unlike, for example, sending a rocket going to the moon, which is a deterministic, directional and goal-oriented process, evolution is a probabilistic process with no direction, no goal. Evolution is blind in the sense that organisms’ survival and reproduction in one generation has no consideration of what would happen in the next generation^1,16^.
Fitness: Fitness is defined as the average number of progenies produced per individual per generation. Fitness can be defined in terms of genes or genotypes^16^.
Genetic assimilation: A process whereby a trait originally appearing in response to an environmental condition becomes genetically encoded by artificial or natural selection. Despite its superficial similarity it is not Lamarckian inheritance^13^.
Genetic hitch hike: Associated response in the frequency of an allele due to its close linkage to advantageous or disadvantageous alleles at neighboring loci^8^.
Historical contingency: Historical events, physical or biological, can affect the genetic/biological composition of populations/ecosystems and determine the subsequent course of evolutionary process and outcome^1,2^.
Homeostasis: Ability of an individual, plants or animals, to maintain internal stability despite being subjected to challenges from the environment^14^.
Molecular complexity: Complexity defined in terms of gene-gene interaction and gene networks for a physiological function, trait or organism^30,54^.
Molecular contingency: Availability of key mutations or variation during evolution can affect rates and outcomes of evolutionary change^2^.
Molecular evolution: Evolution at the level of DNA, RNA and proteins^9^.
Molecular Redundancy: Molecular or genetic information that is in excess of what is the necessary minimum. Redundancy allows phenotypic flexibility.
Necessary Complexity: Minimum number of gene-gene interactions and minimum biochemical path lengths necessary for a given molecular function, trait, or organism.
Neutral evolution: Neutral evolution refers to the rate of molecular evolution of a neutral or nearly neutral allele which is the same as the mutation rate of the allele^9^.
Norm of Reaction: The range of phenotypic expressions of a genotype in a range of environments^15,17^.
Phenotypic plasticity: Phenotypic variation in trait arising as a result of developmental plasticity, molecular redundancy, or gene-environment interactions.^11,15,19^
Pleiotropy: Secondary, side effect of an artificially selected gene in addition to the main effect in a trait^8,16^.
Risk Factor: A segregating allele or a de novo mutation, occurring as sequence variation, deletion, duplication, copy number variation, etc., that disposes its carriers to a disease.
Selective sweep: Rapid evolution of advantageous mutations that drag alleles at closely linked loci leading to loss of sequence variation around the selected locus.
Unnecessary complexity: Gene-gene interactions and biochemical path lengths that are non-essential and over and above the necessary minimum for a given function, trait or organism. Unnecessary complexity is the result of molecular constraints, historical circumstances, and the blind nature of the evolutionary forces.

The network complexity and the ever-increasing number of genes known to affect a given disease pose challenges to estimating the odds of developing a disease for a given set of risk factors or of pinpointing the molecular basis of diseases such as cancer. As a way forward, we can provide some obvious suggestions. First, large-scale genomic studies linked with analytical power of bioinformatics^60,61^ would provide knowledge of variant-specific phenotypic and environmental information. Second, in genomics-based predictive medicine consideration should be given to the estimation of genic heterogeneity/heterozygosity in the network/pathway/cell/tissues as, in the absence of the knowledge of which genes or gene interaction silence a risk factor, the level of heterozygosity can be used as an indicator of the risk-factor’s silence. Third, cancer genomics will benefit by producing ‘functional encyclopedia’ and ‘biochemical pathways atlas’ for organ-specific cancers including cancer subtypes. Finally, basic research in molecular biology has been based on the premise that ‘one doesn’t know what’s abnormal unless one finds out about what’s normal’. Now it is in the interest of the basic sciences to reverse the premise: ‘one can know what’s normal by studying what’s abnormal’. We can live the Hegelian dream and we can show the artificiality of hard divisions between basic and applied sciences.

### Reporting summary

Further information on experimental design is available in the Nature Research Reporting Summary linked to this article.

## Supplementary information

Reporting Summary
